# Comparative proteomics analysis of peanut roots reveals differential mechanisms of cadmium detoxification and translocation between two cultivars differing in cadmium accumulation

**DOI:** 10.1186/s12870-019-1739-5

**Published:** 2019-04-11

**Authors:** Rugang Yu, Qun Jiang, Chen Xv, Lien Li, Sijia Bu, Gangrong Shi

**Affiliations:** College of Life Sciences, Huaibei Normal University, Huaibei, Anhui 235000 People’s Republic of China

**Keywords:** Peanut, Cultivar difference, Cadmium accumulation, Root proteomics

## Abstract

**Background:**

Peanut is one of the most important oil and protein crops, and it exhibits wide cultivar variations in shoot Cd accumulation ability. However, the mechanism of Cd accumulation in peanut shoots has not been well understood. In this study, the root proteomics of two cultivars differing in seed Cd accumulation, Fenghua 1 (F, low Cd cultivar) and Silihong (S, high Cd cultivar), were investigated under 0 (CK) and 2 μM Cd conditions.

**Results:**

A total of 4676 proteins were identified by proteomics screening. Of them, 375, 1762, 1276 and 771 proteins were identified to be differentially expressed proteins (DEPs) for comparison of F_Cd_/F_CK_, S_Cd_/S_CK_, F_CK_/S_CK_ and F_Cd_/S_Cd_, respectively. Silihong is more sensitive to Cd exposure than Fenghua 1 in terms of root proteomics. A total of 30 and 86 DEPs were identified to be related with heavy metal transport and cell wall modification, respectively. The up-regulation of ABCB25, ABCC14, ABCC2, PDR1 and V-ATPases by Cd exposure in Silihong might enhance vacuolar sequestration of Cd and its efflux from symplast to apoplast. The higher Cd accumulation in the root CWs of Silihong might be resulted from its higher capability of CW modification, in which many proteins such as IRX10L, BGLU12-like, BGLU42, EXLB1, XTH30, XTH6, XYL7, PAL3, COMT, CAD1, and CCR1 were involved.

**Conclusions:**

The vacuolar sequestration and efflux of Cd as well as its adsorption in CW might be the principal mechanism of cadmium detoxification in Silihong. The higher capacity of Cd accumulation and translocation of Silihong is an inherent characteristics in which ACA8 and ZIP1 might be involved.

**Electronic supplementary material:**

The online version of this article (10.1186/s12870-019-1739-5) contains supplementary material, which is available to authorized users.

## Background

Cadmium (Cd) is one of the most toxic heavy metals that is extensively discharged into farmlands mainly by anthropogenic activities such as sewage sludge, waste incineration, and phosphate fertilizer [1]. Contamination of soil with Cd directly leads to its overaccumulation in the edible parts of crops, resulting in health risks to human beings through food chains. Various strategies have been applied to reduce the potential risks of Cd pollution to human beings [2]. Among them, screening or breeding low-Cd accumulation cultivar would be a potential way for safe food production in agriculture [3]. This is required to fully elucidate the molecular mechanisms underlying Cd uptake, translocation and accumulation in plants.

Peanut (*Arachis hypogaea* L.) is one of the most important oil crops in the world, and it is also a supplementary food due to its high nutrition. Peanut has a high Cd enrichment capacity in vegetative organs and seeds, showing a wide variation among cultivars [3–7]. The Cd concentration in peanut seeds mainly determined by the attenuation of Cd by high biomass of vegetative tissues and Cd-binding proteins in seeds [3, 5]. It was found that seed Cd concentrations positively correlate with shoot Cd concentrations of seedling, suggesting that shoot Cd concentrations at the vegetative growth stage may be important for determining cultivar differences in Cd accumulation in the seed [3, 5]. Higher proportion of Cd in the soluble fraction (mainly in vacuoles) was observed in the roots of low Cd accumulating cultivars that may contribute to low Cd accumulation in their shoots [8]. Additionally, Cd accumulation in peanuts was shown to associate with root morphology (i.e. high Cd cultivars have longer fine roots than low Cd cultivars) [7]. Although these studies have revealed some physiological mechanisms underlying the cultivar variation in Cd accumulation in peanut, the molecular mechanism has not yet been well investigated.

The accumulation of Cd in plant shoots is controlled by several processes such as Cd apoplastic influx into root tissues, cell wall adsorption, energy-driven transport to cytoplasm across membrane, vacuolar sequestration and xylem loading [9, 10]. Most of these processes are mediated by several families of metal transporters, such as natural resistance associated macrophage proteins (Nramp) [11], zinc/iron transporters (ZIP-IRT) [12] and P1B-ATPases [13, 14]. The overexpression of plasma membrane-localized *OsNramp1* in transgenic rice lines enhanced Cd accumulation in the shoots, which was suggested to involve in cellular Cd uptake [11]. The plasma membrane-localized OsHMA2 [13] and AtHMA4 [14] have been illustrated to be involved in xylem loading of Cd. Cell wall (CW) modification can alter the binding capacity of Cd, and therefore might be responsible for the cultivar difference in root-to-shoot Cd translocation [15, 16]. The pectin and hemicellulose components of the plant CW can offer carboxylic functional groups to join Cd^2+^ and limit Cd transport into root cell [10]. The OsPME14 [17] and AtXTH31 [18] have the ability to increase aluminium (Al) retention in the root CW, which may reduce the Al translocation to shoots. In addition, low molecular weight compounds such as glutathione (GSH), phytochelatins (PCs) and metallothioneins (MTs) have been confirmed to be involved in the vacuolar sequestration of Cd [19].

Proteomic analysis offers accurate information about molecular changes within a range of biological processes, and is a powerful tool for identifying the main regulators in the considered pathways [20]. Up to date, cultivar differences of proteomics in response to Cd stress have been investigated in several plant species such as amaranth [20], *Sedum alfredii* [21], rice [22] and soybean [23]. Some differential expressed proteins (DEPs) associated with vacuolar Cd sequestration, cell wall metabolism, Cd-stress defense and other processes were identified [20, 21]. In peanut, proteomics approach has been applied to address biochemical and physiological effects in response to drought stress [24]. However, little information is available on the differential responses of proteomics to Cd exposure between peanut cultivars.

In this study, based on isobaric tagging for relative and absolute quantification (iTRAQ), a comparative proteomics analysis was conducted on the roots of two peanut cultivars differing in seed Cd accumulation, Fenghua 1 (low Cd cultivar) and Silihong (high Cd cultivar). The main objectives were: (i) to assess root proteome changes in Fenghua 1 and Silihong under different Cd exposures; (ii) to obtain the expression and functional modes of differential expressed proteins in the roots of Fenghua 1 and Silihong under different Cd exposures; and (iii) to elucidate the crucial processes that are responsible for the difference in Cd accumulation between the two cultivars. The results obtained from the present study would contribute to understanding the molecular mechanism of cultivar variation in Cd accumulation of peanuts and provide valuable information for screening or breeding of low Cd cultivars.

## Results

### Cd accumulation and translocation in two peanut cultivars

**Fig. 1:**
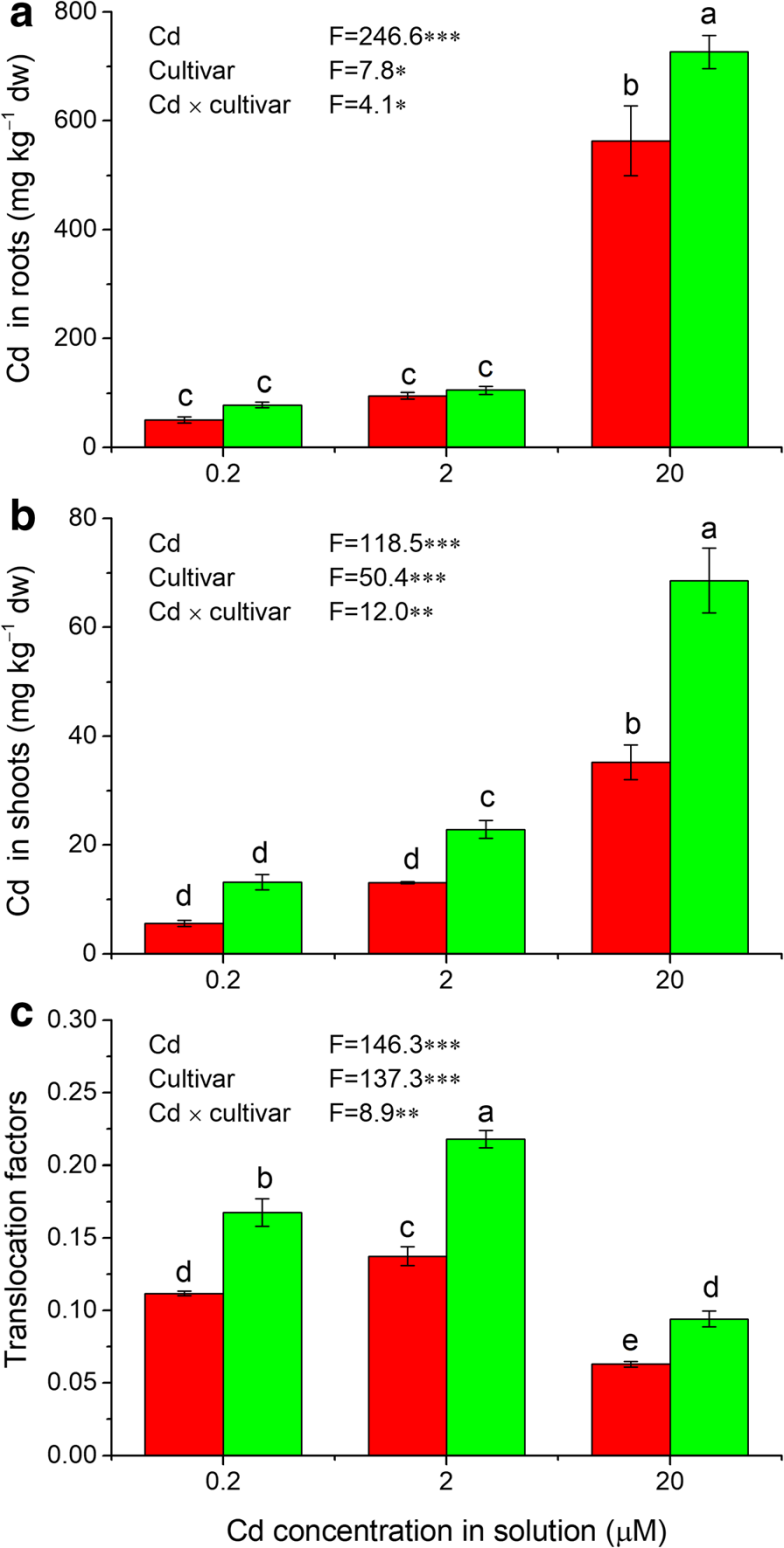
Cd accumulation and translocation in plants of Fenghua 1 (red columns) and Silihong (green columns) exposed to 0.2, 2, and 20 μM Cd for seven days. Different letters above error bars indicate values (mean ± SE, *n* = 3) are significantly different according to Duncan’s test at 0.05 the level. Results of two-way ANOVA were also presented. * *P* < 0.05, ** *P* < 0.01, *** *P* < 0.001

Cd concentrations in roots and shoots as well as translocation factors of Cd from the roots to shoots differed between the two cultivars tested, and were significantly affected by Cd treatments as well as Cd × cultivar interactions (Fig. 1). Cd concentrations in roots and shoots increased with increasing Cd concentrations in the nutrition solutions (Fig. 1a and b), while translocation factors were highest in the 2 μM Cd treatment for both cultivars (Fig. 1c). By contrast, Silihong showed higher shoot Cd concentrations and IFs than Fenghua 1 in all Cd treatments (Fig. 1b and c). Cd concentrations in roots were also observed to be higher in Silihong than in Fenghua 1, depending on Cd treatments (Fig. 1a). The data presented here, concurred with our previous results [5], suggesting that Fenghua 1 has a lower capacity for Cd translocation and accumulation than Silihong.

### Cd, total sugar and uronic acid in root CW components of Fenghua 1 and Silihong

**Fig. 2:**
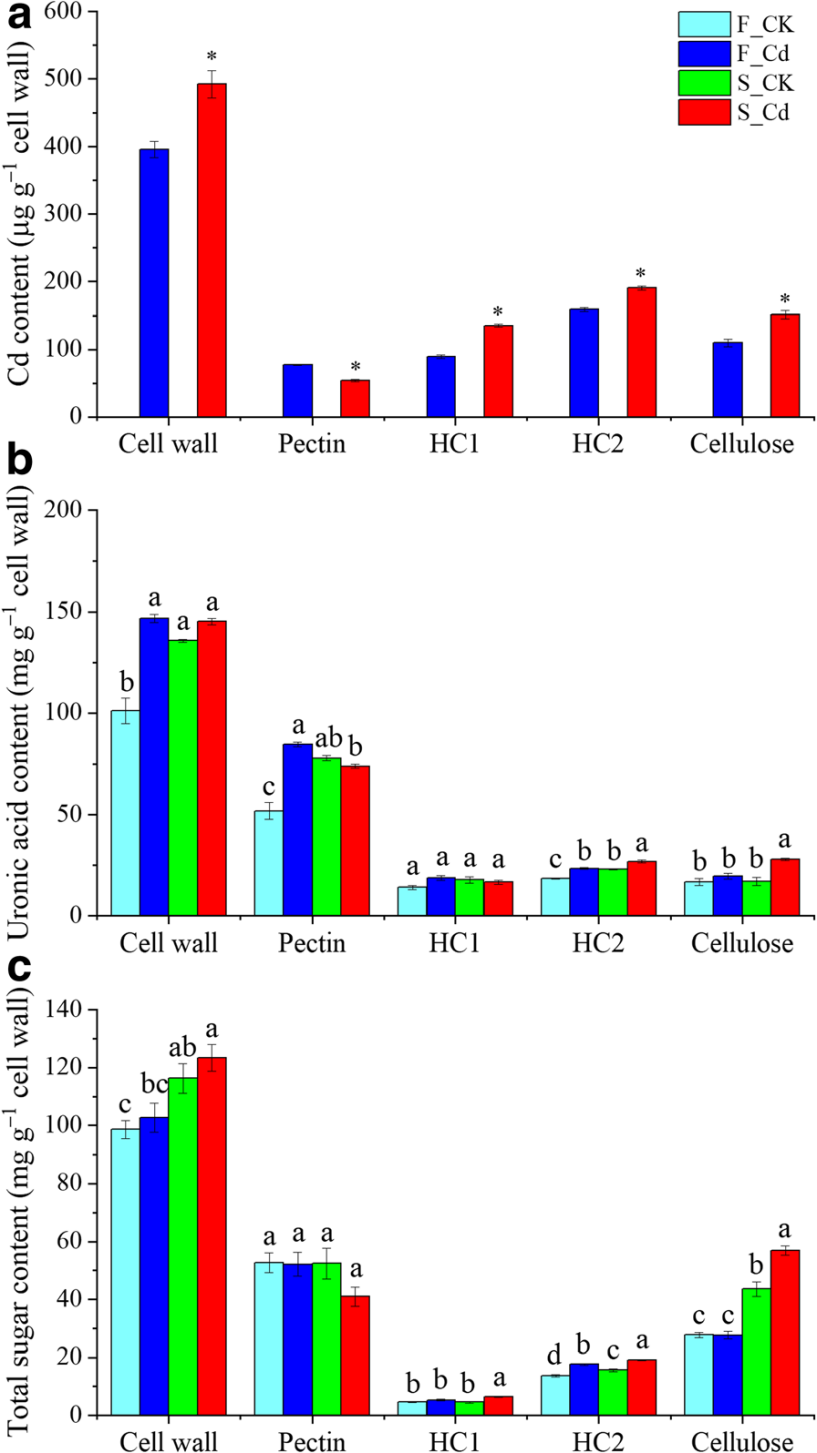
Cd accumulation (**a**) and the content of uronic acid (**b**) and total sugar (**c**) in the cell wall and its components. Asterisk (*) above error bars indicate values (mean ± SE, *n* = 4) are significantly different between Fenghua 1 and Silihong according to independent-samples t-test at 0.05 the level. Different letters indicate values are significantly different according to Duncan’s test at 0.05 the level

The two cultivars differed from each other in Cd concentrations and contents of total sugar and uronic acid in the CW composition (Fig. 2). When plants were exposed to 2 μM Cd, Silihong had higher Cd concentrations in the CW as well as in the hemicelluloses (HC1 and HC2) and cellulose than Fenghua 1, whereas Cd concentrations in the pectin were higher in Fenghua 1 than in Silihong (Fig. 2a). In the CW, most Cd was bound to the hemicelluloses, accounting for 57 and 61% for Fenghua 1 and Silihong respectively. Cd exposure significantly increased the uronic acid content in the CW and its pectin and HC2 components in Fenghua 1, while in Silihong, Cd enhanced the uronic acid content in the HC2 and cellulose (Fig. 2b). Cd did not change the total sugar content in the CW and its pectin component for both cultivars (Fig. 2c). The total sugar content of the hemicelluloses (HC1 and HC2) and cellulose was increased by Cd treatments in Silihong, while they remained unaffected in Fenghua 1 (Fig. 2c). Under Cd exposure, Silihong showed a higher uronic acid content in the HC2 and cellulose and a higher total sugar content in the CW and its hemicelluloses (HC1 and HC2) and cellulose components than Fenghua 1, whereas Fenghua 1 had higher uronic acid contents in the pectin than Silihong (Fig. 2, b and c).

### Overview of protein identified in Fenghua 1 and Silihong

**Fig. 3:**
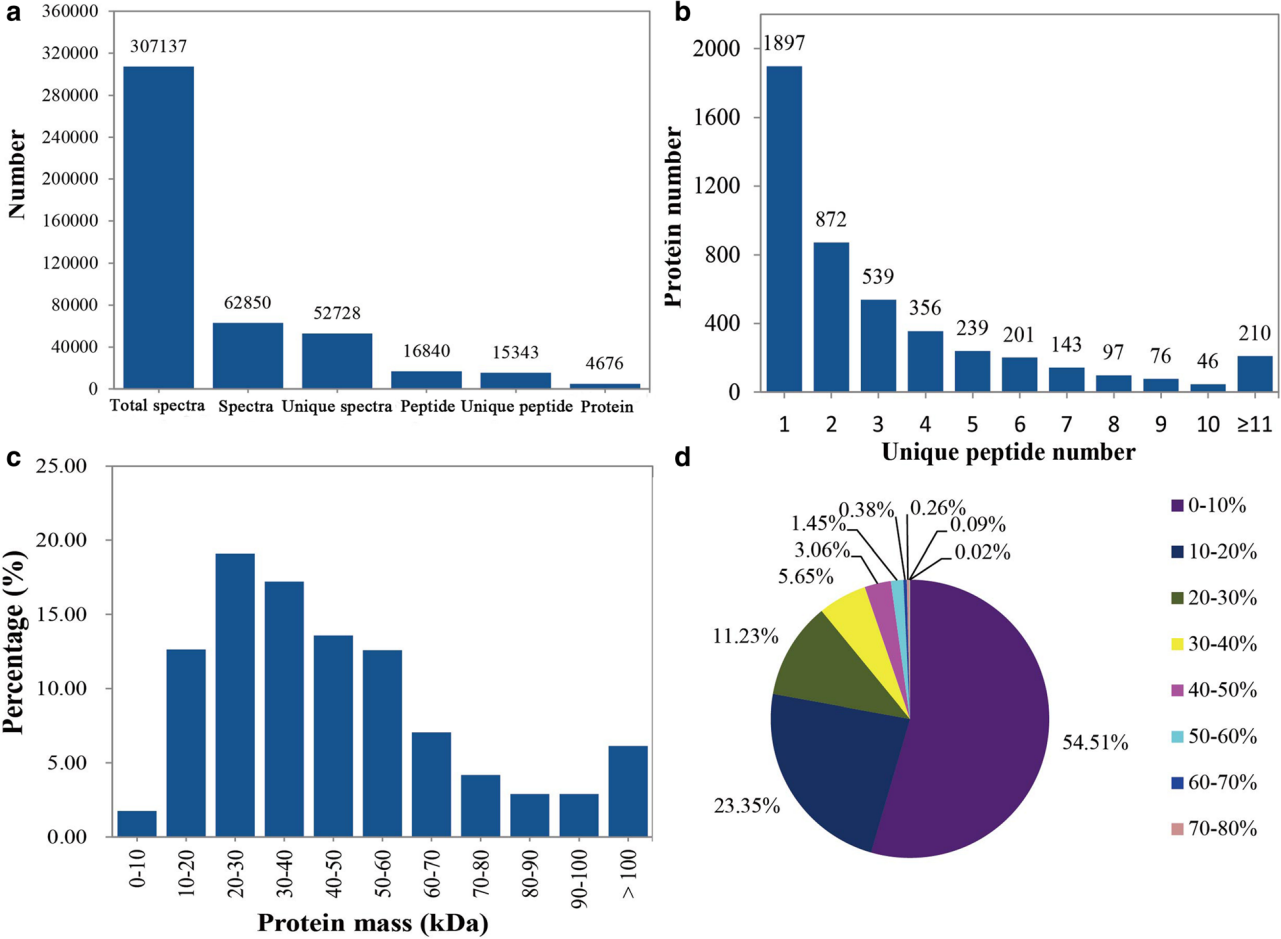
Summary of iTRAQ results. (**a**) total spectra, matched spectra, matched peptides, unique peptides, and identified proteins; (**b**) Number of peptides associated with identified proteins; (**c**) protein relative molecular mass distribution; (**d**) sequence coverage for identified proteins

A total of 307,137 MS/MS spectra from the eight tested samples (two replicates × two species × two treatments) were identified, in which 62,850 spectra were matched with the known spectra (Fig. 3a). Among them, 52,728 unique spectra were matched to 15,343 unique peptides, and 4676 proteins were successfully identified with the cutoff Mascot Percolator FDR ≤ 0.01. The number of proteins with a single peptide, 2–5 peptides, 6–10 peptides and > 11 peptides were 1897, 2006, 563 and 210, respectively (Fig. 3b). The majority of identified protein masses distributed from 11 to 100 kDa with good average coverage. Among them, 11–50 kDa covered 62.53%, 51–100 kDa made up 29.58%, and > 100 kDa comprised 6.14% of the proteins (Fig. 3c). The identified proteins with peptide coverage more than 10 and 20% are 2127 and 1035, accounting for 45.49 and 22.13% respectively (Fig. 3d). The repeatability analysis based on coefficient of variation (CV) was shown in Additional file 1: Figure S1. The lower CV of replicates in the same treatment (0.056–0.086) than that of different treatments (0.10–0.13) indicates a good reproducibility of the data in this study.

### Identification of differentially expressed proteins (DEPs)

**Fig. 4:**
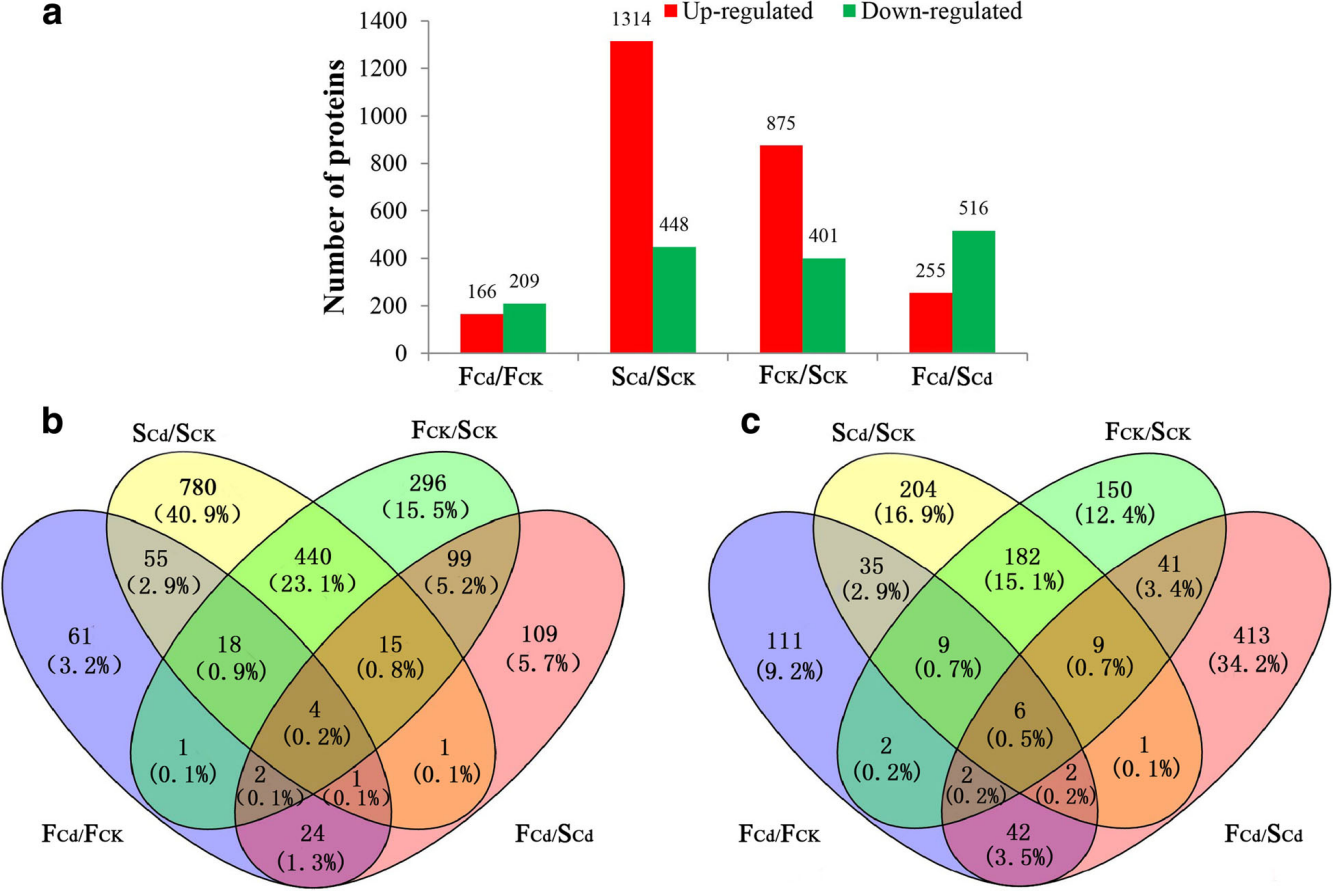
The number of DEPs between pairwise of F_CK_, F_Cd_, S_CK_ and S_Cd_ groups (**a**). Venn diagrams analysis of up- (**b**) and down-regulated (**c**) proteins in any two groups. Each group had two replicates

Based on the standard that Bonferroni-corrected *P*-value < 0.05 and fold changes > 1.2 or < 0.833, a total of 2669 DEPs were identified in the four comparisons (Additional file 2: Table S1). Among them, 375 and 1762 proteins were Cd-responsive DEPs for Fenghua 1 and Silihong, respectively. Cd exposure up-regulated 166 and 1314 proteins for Fenghua 1 and Silihong, respectively, whereas 209 and 448 proteins in the roots of Fenghua 1 and Silihong were down-regulated by Cd (Fig. 4a). Two cultivars share 78 up- and 52 down-regulated Cd-responsive DEPs (Fig. 4b and c). In the control, 1276 DEPs (875 up- and 401 down-regulated proteins) were identified between the two cultivars, while in the Cd treatment, 771 DEPs (255 up- and 516 down-regulated proteins) were identified (Fig. 4a). Regardless of Cd exposure, the abundance of 120 proteins was higher in Fenghua 1 than in Silihong, while those of 58 proteins were higher in Silihong than in Fenghua 1 (Fig. 4b and c).

### GO and KEGG pathway enrichment analysis of the DEPs

A total of 63, 171, 47 and 200 enriched GO terms (*P*-value < 0.05) were obtained from F_Cd_/F_CK_, S_Cd_/S_CK_, F_CK_/S_CK_ and F_Cd_/S_Cd_, respectively, which represent three categories (cellular component, molecular function and biological process) (Additional file 3: Table S2). The most enriched GO terms (*P*-value < 0.01) were listed in Additional file 4: Table S3. For Silihong, the most enriched GO terms of Cd-responsive DEPs include two subcategories of cellular component (nucleus and phragmoplast), 17 subcategories of molecular function (i.e. heterocyclic compound binding, organic cyclic compound binding, etc.), and 15 subcategories of biological process (i.e. cellular process, cellular metabolic process, etc). Cd-responsive DEPs of Fenghua 1 were assigned to molecular function (i.e. glutathione transferase activity) and biological processes (i.e. response to stress, response to oxidative stress) (Additional file 4: Table S3). For DEPs between Fenghua 1 and Silihong, 11 GO terms of cellular component (i.e. plastid, chloroplast and cell wall), 22 GO terms of molecular function (catalytic activity, ion binding, organic cyclic compound binding and cation binding), and 47 GO terms of biological process (metabolic process, response to cadmium ion, response to metal ion and organic acid metabolic process) were most enriched under Cd exposure (Additional file 4: Table S3).

With *P*-value < 0.05 as the cutoff, a total of 7, 8, 5 and 16 significantly enriched pathways were identified in F_Cd_/F_CK_, S_Cd_/S_CK_, F_CK_/S_CK_ and F_Cd_/S_Cd_, respectively (Table 1). For Cd-responsive DEPs, the top two significantly enriched pathways were ‘phenylpropanoid biosynthesis’ [ko00940] and ‘Glutathione metabolism’ [ko00480] in Fenghua 1, and ‘Spliceosome’ [ko03040] and ‘Pyruvate metabolism’ [ko00620] in Silihong. Moreover, only one pathway, ‘glutathione metabolism’, was significantly enriched in both cultivars. For DEPs between Fenghua 1 and Silihong under Cd exposure, the most predominant pathways were ‘metabolic pathway’ [ko01100], ‘biosynthesis of secondary metabolites’ [ko01110], ‘Carbon metabolism’ [ko01200], ‘phenylpropanoid biosynthesis’ and ‘biosynthesis of amino acids’ [ko01230].

**Table 1:** Significantly enriched KEGG pathways for the DEPs

Pathway	Sample number				*P*-value				Pathway ID
	F_Cd_/F_CK_	S_Cd_/S_CK_	F_CK_/S_CK_	F_Cd_/S_Cd_	F_Cd_/F_CK_	S_Cd_/S_CK_	F_CK_/S_CK_	F_Cd_/S_Cd_	
Glutathione metabolism	11	35	13	19	0.0156	0.0239	0.9609	0.0178	ko00480
Phenylpropanoid biosynthesis	20	55	55	53	0.0085	0.6295	0.0038	0.0000	ko00940
Carotenoid biosynthesis	6	13	5	2	0.0125	0.1544	0.8753	0.9500	ko00906
Phosphatidylinositol signaling system	5	7	6	2	0.0158	0.7016	0.4729	0.8767	ko04070
Photosynthesis	6	5	4	6	0.0248	0.9975	0.9796	0.3996	ko00195
Inositol phosphate metabolism	6	6	6	4	0.0381	0.9967	0.9193	0.8351	ko00562
Cutin, suberine and wax biosynthesis	3	3	2	2	0.0474	0.8587	0.8450	0.5795	ko00073
Spliceosome	8	55	40	13	0.5496	0.0012	0.0062	0.9105	ko03040
Benzoxazinoid biosynthesis	1	5	1	4	0.3833	0.0340	0.8515	0.0092	ko00402
Terpenoid backbone biosynthesis	1	18	6	10	0.9184	0.0203	0.8861	0.0271	ko00900
Stilbenoid, diarylheptanoid and gingerol biosynthesis	2	9	6	4	0.2070	0.0042	0.0505	0.1001	ko00945
Ribosome biogenesis in eukaryotes	1	16	8	6	0.8560	0.0046	0.3181	0.2100	ko03008
Citrate cycle (TCA cycle)	1	24	4	9	0.9759	0.0390	0.9996	0.3754	ko00020
Pyruvate metabolism	2	38	10	17	0.9855	0.0314	0.9993	0.1453	ko00620
Mismatch repair	1	7	7	1	0.6199	0.1311	0.0226	0.8926	ko03430
Plant-pathogen interaction	6	38	31	18	0.6645	0.1578	0.0437	0.1951	ko04626
Photosynthesis - antenna proteins	–	5	5	1	–	0.0814	0.0187	0.7277	ko00196
Biosynthesis of secondary metabolites	66	279	173	175	0.0811	0.5340	0.9913	0.0000	ko01110
Metabolic pathways	101	446	293	271	0.2113	0.9448	0.9988	0.0000	ko01100
Alanine, aspartate and glutamate metabolism	4	13	6	13	0.2843	0.6237	0.9438	0.0033	ko00250
Cyanoamino acid metabolism	4	8	11	12	0.3746	0.9964	0.5438	0.0288	ko00460
Carbon fixation in photosynthetic organisms	5	17	12	17	0.4541	0.9323	0.8876	0.0109	ko00710
Nitrogen metabolism	2	8	7	8	0.4914	0.5933	0.3383	0.0169	ko00910
Pentose phosphate pathway	4	17	14	17	0.5801	0.8401	0.5716	0.0039	ko00030
Aminoacyl-tRNA biosynthesis	3	18	10	16	0.7672	0.7244	0.9212	0.0079	ko00970
Biosynthesis of amino acids	12	75	28	51	0.7999	0.3595	1.0000	0.0002	ko01230
Carbon metabolism	11	81	37	53	0.9429	0.5059	0.9997	0.0011	ko01200
One carbon pool by folate	–	7	2	7	–	0.6400	0.9808	0.0302	ko00670
Arginine and proline metabolism	–	11	3	10	–	0.7399	0.9969	0.0337	ko00330

### DEPs related to heavy metal transport

A total of 79 DEPs were identified to have homology with various transporters (Additional file 5: Table S4). Among them, 30 DEPs were identified to be related with heavy metal transport, including ATP-binding cassette (ABC) transporters, zinc transporter (ZIP1), metal tolerance protein (e.g. MTPc2 and MTPc4), oligopeptide transporter 7 (OPT7), pleiotropic drug resistance protein 1 (PDR1), V-type proton ATPase (V-ATPase), endoplasmic reticulum-type calcium-transporting ATPase 4 (ECA4) and plasma membrane-type calcium-transporting ATPase 8 (ACA8) (Table **2**). Most of heavy metal transporters (20 proteins) were altered by Cd exposure in Silihong, including 15 up-regulated proteins (ABCB11, ABCB25, ABCC4, ABCC2, ABCC14, ABCG8-like, ABCF1, ABCF3, ECA4, MTP2, PAA1, V-ATPase subunit H, V-ATPase subunit d2, V-ATPase subunit H isoform X3) and 5 down-regulated proteins (ATX1, ZIP1, ACA8, V-ATPase subunit D). In contrast, only two heavy metal transporters were identified to be Cd-responsive DEPs in Fenghua 1, including a up-regulated protein (ABCG8) and a down-regulated protein (OPT7) (Table **2**). Under Cd exposure, 11 heavy metal transporters (ABCB11, ABCB25, ABCC14, PDR1, ECA4, PAA1, MTPc2, MTPc4, V-ATPase catalytic subunit A) showed higher expression in Silihong than in Fenghua, while V-ATPase subunit G and V- ATPase 16 kDa proteolipid subunit isoform X4 were lower in Silihong than in Fenghua. All these proteins were not DEPs of the two cultivars in the absence of Cd (Table **2**).

**Table 2:** DEPs involved in metal transport in roots of the two peanut cultivars

Protein ID	Protein name	Abbr.	Mean Ratio			
			F_Cd_/F_CK_	S_Cd_/S_CK_	F_CK_/S_CK_	F_Cd_/S_Cd_
XP_015967663.1	ABC transporter A family member 2	ABCA2	0.85	1.10	1.28^*^	0.91
XP_015967661.1	ABC transporter A family member 7 isoform X2	ABCA7	0.86	1.12	1.22^*^	0.94
XP_020982984.1	ABC transporter B family member 11	ABCB11	1.01	1.79^*^	1.00	0.57^*^
XP_015939460.1	ABC transporter B family member 11	ABCB11	1.12	1.71^*^	1.16	0.81^*^
XP_015931717.1	ABC transporter B family member 25, mitochondrial	ABCB25	0.98	1.38^*^	1.11	0.80^*^
XP_015959705.1	ABC transporter C family member 14	ABCC14	1.01	1.65^*^	1.17	0.72^*^
XP_015942829.1	ABC transporter C family member 2-like	ABCC2	0.96	1.27^*^	1.17	0.87
XP_015951104.1	ABC transporter C family member 4	ABCC4	0.94	1.34^*^	1.25^*^	0.86
XP_015935759.1	ABC transporter F family member 1	ABCF1	1.05	1.51^*^	1.24^*^	0.90
XP_015938024.1	ABC transporter F family member 3	ABCF3	1.02	1.33^*^	1.15	0.89
XP_020986785.1	ABC transporter G family member 8-like	ABCG8	1.30^*^	1.48^*^	1.03	0.92
XP_015969915.1	calcium-transporting ATPase 4, endoplasmic reticulum-type	Ca^2+^-ATPase4	1.12	1.69^*^	1.11	0.76^*^
XP_020993898.1	calcium-transporting ATPase 8, plasma membrane isoform X6	Ca^2+^-ATPase8	1.43	0.74^*^	0.65^*^	1.26
XP_015941278.1	copper transport protein ATX1	ATX1	0.79	0.64^*^	0.72^*^	0.89
XP_015949536.1	copper transport protein ATX1	ATX1	1.05	0.79^*^	0.74^*^	1.02
XP_015943398.1	copper-transporting ATPase PAA1, chloroplastic	PAA1	1.02	1.24^*^	1.00	0.81^*^
XP_015951091.1	metal tolerance protein 2 isoform X2	MTP2	1.61	2.19^*^	0.68^*^	0.54
XP_015969432.1	metal tolerance protein C2	MTPc2	0.80	1.22	1.24	0.80^*^
XP_015938018.1	metal tolerance protein C4	MTPc4	0.84	1.18	1.07	0.77^*^
XP_015951854.1	oligopeptide transporter 7	OPT7	0.77^*^	0.94	1.13	0.91
XP_015937455.1	pleiotropic drug resistance protein 1	PDR1	0.89	1.28	1.05	0.75^*^
XP_020983616.1	V-type proton ATPase 16 kDa proteolipid subunit isoform X4	V-ATPase X4	1.00	0.88	1.16	1.28^*^
XP_015958526.1	V-type proton ATPase catalytic subunit A	V-ATPase subunit A	1.00	1.19	0.95	0.80^*^
XP_015960547.1	V-type proton ATPase subunit D	V-ATPase subunit D	1.09	0.82^*^	0.83^*^	1.07
XP_015962803.1	V-type proton ATPase subunit d2	V-ATPase subunit d2	1.01	1.41^*^	1.09	0.81^*^
XP_020999809.1	V-type proton ATPase subunit F	V-ATPase subunit F	0.92	1.23	1.33^*^	1.02
XP_020986386.1	V-type proton ATPase subunit G	V-ATPase subunit G	1.05	0.86	1.04	1.27^*^
XP_015964081.1	V-type proton ATPase subunit H	V-ATPase subunit H	1.04	1.33^*^	1.04	0.85
XP_015948413.1	V-type proton ATPase subunit H isoform X3	V-ATPase subunit HX3	1.07	1.44^*^	1.16	0.89
XP_020984784.1	zinc transporter 1	ZIP1	0.97	0.78^*^	0.73^*^	0.91

* indicate the protein is differentially expressed in the pairwise comparison

### DEPs related to cell wall modification

Totally, 86 DEPs involved in CW modification were identified, including 66 proteins related to the CW degradation, 14 proteins related to CW synthesis and six expansin proteins (EPs) (Additional file 6: Table S5). Cd increased the abundance of 11 proteins belonging to pectinesterase/pectinesterase inhibitors (PPE8B-like, PPE51), xyloglucan endotransglucosylase/hydrolase proteins (XTH1, XTH23), cytochrome P450, beta-glucosidase (GLUC45), endoglucanase 9 (EG9), pectin acetylesterase (PAE3), pectinesterase (PE2), endochitinase and glucan endo-1,3-beta-glucosidase (XP_015952748.1), but decreased that of casparian strip membrane protein 2, glucan endo-1,3-beta-glucosidase (XP_015969184.1), PE (XP_020993886.1), expansin-like B1, and XTH22 in Fenghua 1. In Silihong, 32 proteins belonging to GLUCs, EGs, XTHs, P450, PEs, PPE, expansins, beta-galactosidase (β-Gal), polygalacturonases (PGs), pectate lyases (PLs), phospholipase A-2-activating proteins (PLAP), beta-1,4-xylosyltransferase (IRX10L), and endo-1,3;1,4-beta-D-glucanase were up-regulated by Cd exposure, and 12 proteins belonging to XTHs, GLUCs, PEs, P450, PPEs, casparian strip membrane protein 2, chitinase-3-like protein, and expansin-like were down-regulated by Cd exposure. Under Cd exposure, a total of 41 DEPs were identified between the two cultivars. Among them, 31 were found to highly expressed in Fenghua 1 compared with Silihong, while the other ten proteins were lower in Fenghua 1 than Silihong regarding the abundance. Regardless of Cd exposure, 13 proteins including four glucan endo-1,3-beta-glucosidase, three polygalacturonase, as well as PE, *β*-Gal, XTH31, polygalacturonase inhibitor 2 (PGIP2), beta-xylosidase/alpha-L-arabinofuranosidase 2 (XLA2), and endo-1,3;1,4-beta-D-glucanase-like, were higher in abundance in Fenghua 1 than in Silihong, whereas the abundance of four proteins (chitinase-3-like protein 1, expansin-like B1, beta-galactosidase isoform X2 and beta-glucosidase 12-like) were lower in Fenghua 1 than Silihong.

A total of 64 DEPs were identified to be involved in lignin biosynthesis (Additional file 7: Table S6). Compared with the control, Cd exposure up-regulated 8 and 16 proteins for Fenghua 1 and Silihong respectively, while five proteins in Fenghua 1 and 16 proteins in Silihong were down-regulated by Cd. Under Cd exposure, 26 proteins belonging to peroxidases (PODs), laccases (LACs) and caffeoyl-CoA O-methyltransferase (CCoA-OMT) were more abundant in Fenghua 1 than in Silihong, while only five proteins (LAC3, CCR2, 4CL2, HCT, PAL3) were higher in Silihong than in Fenghua 1 (Additional file 7: Table S6).

### Verification of iTRAQ data by RT-qPCR

**Fig. 5:**
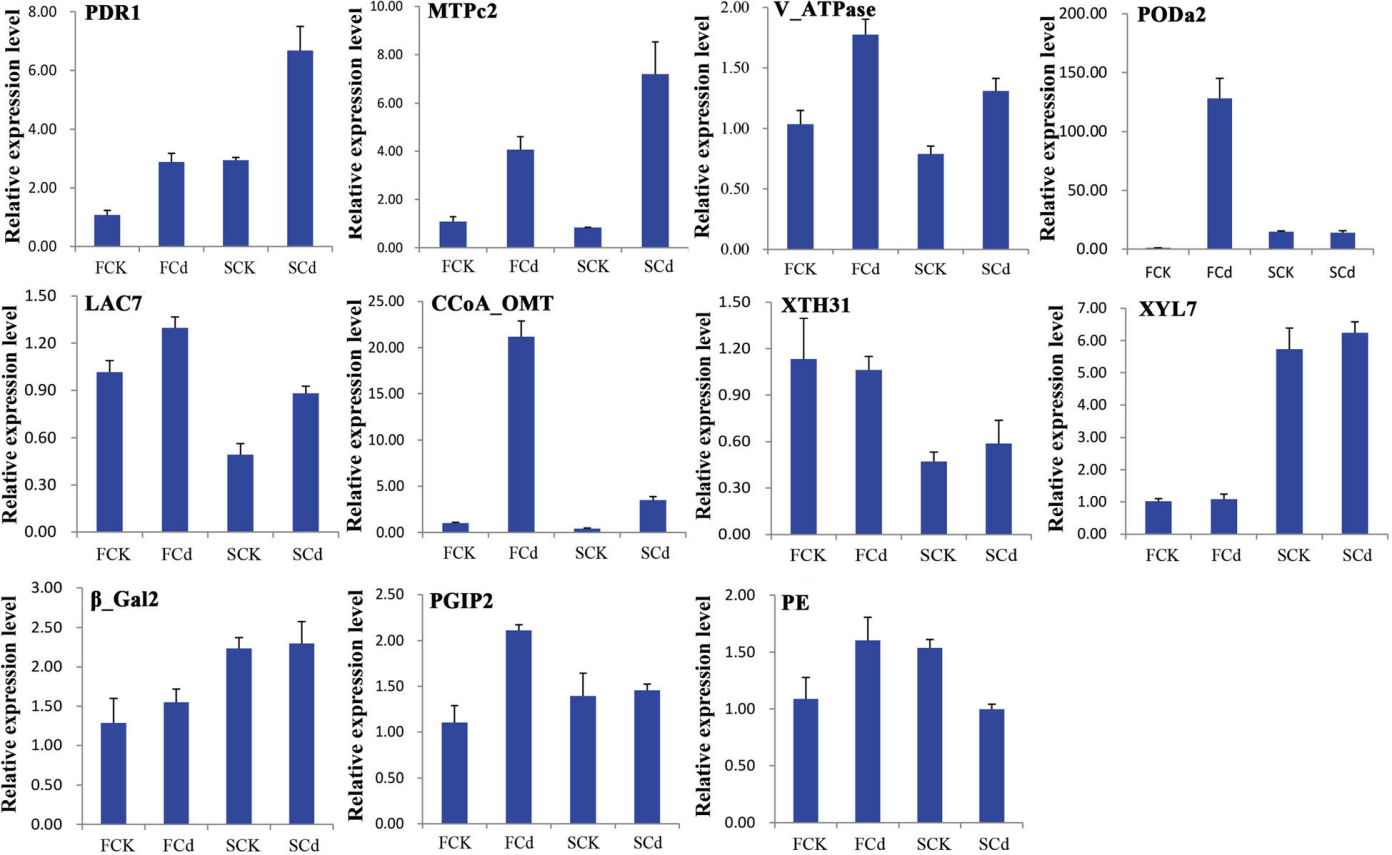
RT-qPCR analyses of 11 candidate DEPs under the control and Cd treatment in roots of two peanut cultivars. Each bar represents the mean ± SD of triplicate assays

To validate the iTRAQ results, the transcriptional analysis of 11 candidate DEPs was performed using RT-qPCR (Fig. 5). The expression level of 11, 10, 8 and 7 genes showed the same or similar trend of change with their corresponding protein abundance in F_Cd_/S_Cd_, S_Cd_/S_CK_, F_CK_/S_CK_ and F_Cd_/F_CK_, respectively (Fig. 5; Additional file 2: Table S1). These results suggested that the majority of proteins were regulated directly at the transcription level. However, four genes encoding peroxidase A2 (F_CK_/S_CK_), pectinesterase and pleiotropic drug resistance protein 1 (F_Cd_/F_CK_, F_CK_/S_CK_) and metal tolerance protein C2 (F_Cd_/F_CK_), indicated an inconsistent relationship between the patterns of mRNA expression and protein abundance (Fig. 5; Additional file 2: Table S1). For instance, the peroxidase A2 was down-regulated at the transcription level in F_CK_/S_CK_, whereas it was obviously up-regulated at the protein level. The pectinesterase and pleiotropic drug resistance protein 1 showed opposing expression pattern in F_Cd_/F_CK_ at mRNA levels and their corresponding protein abundance. These paradoxical discrepancies between RT-qPCR and iTRAQ proteomics might attribute to the sensitivity between two analysis methods and a difference between the processes of mRNA translation and posttranslational modifications.

## Discussion

### Differential responses of proteomic profiles to Cd between Fenghua 1 and Silihong

Proteome studies have been successfully and increasingly used for revealing the mechanism of Cd resistance and accumulation in some plant species [20, 22, 23]. To elucidate the mechanisms involved in cultivar differences in Cd accumulation in peanuts, root proteomic profiles of Fenghua 1 and Silihong were analysed under control and Cd-treated conditions by an iTRAQ-based quantitative proteomics approach. Totally 4676 proteins were identified in this study, from which 375, 1762, 1276 and 771 proteins exhibited significant differential abundance in F_Cd_/F_CK_, S_Cd_/S_CK_, F_CK_/S_CK_ and F_Cd_/S_Cd_ comparison, respectively (Additional file 2: Table S1). Cd induced 375 DEPs in the roots of Fenghua 1, whereas 1762 proteins were identified between 0 and 2 μM Cd treatments in Silihong, which was 3.7-fold higher than those of Fenghua 1 (Fig. 4a). Between the two cultivars, most of DEPs (68.7%) showed higher abundance in Fenghua 1 in the absence of Cd, while in the presence of Cd, most of DEPs (67%) were higher in Silihong (Fig. 4a). These results suggest that the two cultivars differed in the molecular mechanisms in response to Cd exposure. Silihong is more sensitive to Cd exposure than Fenghua 1 in terms of root proteomics.

### Transporters involved in Cd uptake and translocation in Fenghua 1 and Silihong

Transport of Cd^2+^ across the membrane system in plant cells has been shown to be mediated by transports of divalent ions such as Zn^2+^, Ca^2+^, Fe^2+^, Mn^2+^, Cu^2+^ and Mg^2+^ [9]. However, this is varied among plant species or among cultivars/ecotypes within a species, and little is known about the mechanism of Cd uptake and translocation in peanut plants. In the current study, we found that Silihong shows higher shoot Cd concentration and higher translocation factors than Fenghua 1 (Fig. 1). The results were concurred with our previous findings, illustrating that high seed Cd-accumulating cultivars have higher Cd concentrations in shoots of seedlings and vice versa [3, 5].

The root-to-shoot Cd translocation in plants is mainly influenced by several processes, including cell wall adsorption, influx into root cells via the plasma membrane, vacuolar sequestration and xylem loading [9, 10]. Unexpectedly, our results showed that Silihong (high Cd-accumulating cultivar) exhibit higher Cd accumulation in root CWs than Fenghua 1 (low Cd-accumulating cultivar) (Fig. 2). Similar results have been reported by Wang et al. [25] in two contrasting soybean cultivars (HX3 and BX). These findings have refuted our hypothesis that cell wall adsorption might contribute to the difference of Cd accumulation and translocation between Fenghua 1 and Silihong.

Thus, the difference of Cd accumulation between Fenghua 1 and Silihong might be determined by transporter mediated processes. In the current study, 30 DEPs were identified to be involved in heavy metal transport (Table **2**). Among them, 20 heavy metal transporters were found to be Cd-responsive DEPs in Silihong, including 15 up-regulated proteins and 5 down-regulated proteins. In contrast, only two heavy metal transporters were identified to be DEPs in Fenghua 1.

Some members ABC transporters have been verified to be involved in the uptake and translocation of Cd in plants [26]. In this study, eight ABC transporters (ABCB11, ABCB25, ABCC2, ABCC4, ABCC14, ABCG8-like, ABCF1, ABCF3) were found to be induced by Cd in Silihong, while in Fenghua 1 only one ABC transporters (ABCG8) was induced. Moreover, four ABC transporters (ABCB11, ABCB25, ABCC14 and PDR1) showed higher expressions in Silihong than in Fenghua 1 (Table **2**).

ABCB25 of peanuts is homologous with AtABCB27/AtALS1 in *Arabidopsis*, which is a vacuolar membrane localized transporter in vasculature of the root tip and possibly involved in aluminum vacuolar sequestration [27]. ABCC14 is highly homologous to AtABCC14/AtMRP10 (71% identity) and AtABCC4/AtMRP4 (68% identity). AtABCC2 was firstly identified as a vacuolar-localized transporter of PC-As complexes in *Arabidopsis*, and it has been shown to play a role in Cd (and Hg) tolerance by sequestrating Cd in root vacuoles [28]. AtABCC4 was shown to complement yeast *Δycf1* mutant for its Cd hypersensitivity, partially restoring Cd tolerance [29]. PDR1 (XP_015937455.1) is orthologous to AtPDR12/ABCG40, which is a plasma membrane transporter that functions as an efflux pump for Pb^2+^ exclusion in *Arabidopsis* [30]. In cucumber, CsPDR12/CsABCG40 was up-regulated under Cd stress [31]. Thus, the up-regulation of these transporters by Cd exposure in Silihong might enhance Cd detoxification by increasing its vacuolar sequestration or efflux from symplast to apoplast.

MTPs have been reported to mediate Zn/Cd uptake and translocation [32]. In this study, we found that MTP2 were down-accumulated in Cd-exposed plants of Silihong. Moreover, MTPc2 and MTPc4 were also detected to be higher in Silihong than in Fenghua 1. Peanut MTPc2 is homology with AtMTPc2/AtMTP5 in *Arabidopsis*, which was reported to form a heteromeric complex with AtMTP12 to transport Zn into the Golgi apparatus [32]. MTP2 and MTPc4 are homology with AtMTP6 and AtMTPc4/AtMTP7 in *Arabidopsis*, respectively. Nevertheless, characterization of both proteins was not analyzed. Whether these MTPs associate with Cd uptake and translocation in peanut plants or not require further studies.

The V-ATPase a tonoplast-localized proton pump that provides energy for ion transport. It is a multiheteromeric complex of at least 11 different subunits (i.e. atpv A-H, atpv a, c and d) [33]. Antiport activity of Cd across the tonoplast requires a proton gradient, which is largely dependent on the activity of the V-ATPase [33]. In the current study, three proteins of two V-ATPase subunits (subunit H and subunit d2) were induced by Cd in Silihong (Table **2**). These results suggest that V-ATPases may promote Cd into the endomembrane systems in the roots, and consequently, improving Cd resistance and reducing root-to-shoot Cd translocation in peanut plants. Meanwhile, higher abundance of V-ATPase subunit G and V-ATPase 16 kDa proteolipid subunit isoform X4 may be partially responsible for low Cd translocation in Fenghua 1.

It was also observed that two P-type ATPases, Ca^2+^-ATPase 4 and PAA1, were exclusively induced by Cd in Silihong, and their abundance was higher in Silihong than in Fenghua 1 under Cd exposure (Table **2**). Peanut Ca^2+^-ATPase 4 exhibits high sequence similarity to AtECA1, an endoplasmic reticulum (ER)-localized P_2A_-type ATPase in *Arabidopsis* that confers to Ca^2+^/Mn^2+^ transport from the cytosol into the ER [34]. PAA1 is a chloroplast-envelope P_IB_-ATPase that functions in copper transport in chloroplasts [35]. It is unknown whether Ca^2+^-ATPase 4 and PAA1 is involved in Cd transport, however, their up-regulation might improve nutrition of essential nutrients such as Ca, Mn and Cu in Silihong.

In that all abovementioned Cd-responsive transporters could not well explain the difference of Cd accumulation between Silihong and Fenghua 1, we speculate that the mechanism may be the inherent characteristics. Interestingly, we found that several transporters such as ATX1, ACA8, ZIP1 and V-type proton ATPase subunit D were higher in Silihong than in Fenghua 1 under Cd-free conditions. Moreover, all these transporters were suppressed by Cd in Silihong, while they were unchanged in Fenghua 1. ATX1 is a Cu chaperone that delivers Cu and Cd to Ccc2, which is an ER membrane-localized Cu-transporting ATPase [36]. This protein may not be involved in root-to-shoot Cd transport in plants. ACA8 as a P-type ATPase involved in cation efflux from the cytosol [37], has been described to be a candidate for Cd accumulation that possiblely move Cd to the xylem in *Noccaea caerulescens* [38]. ZIP1 of peanut exhibits high sequence similarity to *Arabidopsis* AtZIP2. AtZIP2 is expressed in the plasma membrane of root stele cells [12], and it is likely to be involved in Zn, Mn and Cd translocation from roots to shoots [12, 39]. It seems that higher abundance of ACA8 and ZIP1 in the roots of Silihong contributes to its higher capacity for root-to-shoot Cd translocation.

### Cell wall modifying proteins in roots of Fenghua 1 and Silihong

The CW of roots as the first composition for plants in contact with Cd, play key roles in the prevention of Cd from entering into the cytoplast [9, 10]. CWs contain many negatively charged groups such as –COOH, −OH and –SH, which enable metal cations to bind into CWs effectively [15]. Our proteomic analysis showed that a total of 150 DEPs were involved in the modification of cell walls in the roots of the two peanut cultivars (Additional file 6: Table S5 and Additional file 7: Table S6).

Pectin is mainly responsible for the capacity of CW for the binding of metal cations due to their highly negatively charged carboxylic groups [17]. The accumulation of Al has been reported to positively relate to pectin content but negatively correlate with the degree of methylesterification of pectin in *Zea mays* [40, 41]. Cd induced increases in low-methylesterified pectin were found in flax hypocotyls [42], basket willow [43] and rice [44]. More interestingly, the hyperaccumulating ecotype of *Sedum alfredii* was found to show lower pectin content and activity of pectin methylesterase than the non-hyperaccumulating one, leading to a high methylesterified pectins that may be responsible for higher root-to-shoot Cd translocation of the hyperaccumulating ecotype [15].

In this study, we found that some proteins related to pectin decomposition such as *β*-Gal, PG, and PL1 were significantly induced in Cd-exposed Silihong, while in Fenghua 1, they remained unaffected (Additional file 6: Table S5). The results indicate that Cd might induce a degradation of pectin in the roots of Silihong that may reduce its binding in CWs. Furthermore, higher abundance of PEs, PE8B, PPE61, and PGIP2 in Fenghua 1 (Additional file 6: Table S5) suggests that Fenghua 1 shows a higher capacity for preventing pectin decomposition and methylesterification than Silihong under Cd exposure. This was proven by the higher uronic acid and total sugar contents in pectin of Fenghua 1, which was accompanied with higher pectin Cd contents, compared with Silihong (Fig. 2a).

Hemicellulose and cellulose are major sites binding metal ions in the cell wall [18, 44]. In the nonpoalean monocotyledons and dicotyledons, xyloglucan is the major hemicellulose of the primary CW [18]. Our results indicate that most Cd in the CW is bound to the hemicelluloses for both cultivars (Fig. 2a). Compared with Fenghua 1, Silihong has higher Cd concentrations in the hemicelluloses (HC1 and HC2) and cellulose (Fig. 2a), which is accompanied by a higher total sugar content in hemicelluloses (HC1 and HC2) and cellulose (Fig. 2c). Hence, the higher contents of hemicelluloses and cellulose might be responsible for higher Cd in the CW of Silihong.

The modification of CWs is catalyzed by several enzymes including XTHs and Expansins. XTHs contribute to cell wall extension either by cutting and relinking xyloglucan chains or by catalyzing the hydrolysis of xyloglucan [18]. Expansins can induce loosening and extension of plant CWs by disrupting non-covalent bonding between cellulose microfibrils and matrix glucans. In the present study, we found that two XTH proteins (XTH1 and XTH23) were induced by Cd exposure in Fenghua 1, while in Silihong, five proteins (XTH1, XTH2, XTH6, XTH30, and XTH32) and two α-expansins (EXPA11 and EXPA8) were induced (Additional file 6: Table S5). Moreover, the total sugar content of the hemicelluloses (HC1 and HC2) and cellulose was increased by Cd treatments in Silihong, while they remained unaffected in Fenghua 1 (Fig. 2c). These results indicate that XTHs and α-expansins might be responsible for Cd resistance by maintaining CW extensibility in Silihong under Cd exposure.

Besides XTHs and Expansins, the CW was also modified by other polysaccharide-degrading enzymes, represented by β-glucosidase, glycosyltransferase and endo-xylanases. Compared with Fenghua 1, Silihong showed a higher abundance of IRX10L, BGLU12-like, BGLU42, EXLB1, XTH30, XTH6 and XYL7, but a lower abundance of BGLU13-like, endo-1,3;1,4-beta-D-glucanase-like, XLA1 and XLA2 under Cd exposure (Additional file 6: Table S5). IRX10L is a glycosyltransferase that plays a role in elongation of the xylan backbone in the hemicellulose [45]. BGLUs can break down the terminal, non-reducing β-D-glucosyl residues with release of beta-D-glucose. Endo-1,3-1,4-beta-D-glucanase act on the endohydrolysis of (1–3)- or (1–4)-linkages in β-D-glucans, whereas XYLs are active on these latter oligomers releasing xylose. XLAs are also needed for the degradation of arabinoxylan in that they act synergistically with endoxylanases and cleave arabinose from the backbone. The results presented here suggest that the two cultivars differ from each other in the modification of CWs under Cd exposure. By contrast, more hemicellulose modifying enzymes were observed to show a higher abundance in Silihong compared with Fenghua 1, indicating that it has a higher capacity for CW remodelling. This might better explain the higher contents of hemicellulose and cellulose in the root of Silihong as indicated by the total sugar (Fig. 2c). It seems that the higher Cd accumulation in hemicelluloses and cellulose of the root CWs in Silihong might be resulted from its higher capability of CW modification.

Lignin, a phenolic polymer derived from hydroxycinnamyl alcohols, is also a major factor limiting Cd uptake into root cells [10, 46]. Lignin contains several functional groups such as hydroxyl, phenolic, carboxyl, methoxyl, aldehyde, and benzyl alcohol groups, which is able to bind metals to CWs [47]. Lignin is mainly presented in secondary thickened plant cells, where it is covalently linked to non-cellulosic polysaccharides and provides rigidity and impermeability to CWs [48]. Lignin predominantly derived from three monolignols including p-coumaryl, coniferyl, and sinapyl alcohols, forming p-hydroxyphenyl (H), guaiacyl (G), and syringyl (S) lignin, respectively. These processes were regulated by a large number of enzymes such as PAL, COMT, CAD, CCR, HCT, 4CL, CCoA-OMTs and so on [48].

In the current study, we found that Cd increases the abundance of CCoA-OMT and CAD6 in the roots of Fenghua 1. Both CCoA-OMT and CAD6 are key enzymes for the synthesis of guaiacyl lignin [48]. In contrast, almost all enzymes related to the synthesis of lignin monolignols were significantly induced by Cd in Silihong, including PAL3, COMT, CAD1, two CCR1, two HCT, three 4CLs, and three CCoA-OMTs (Additional file 7: Table S6). In comparison with Fenghua 1, Silihong shows higher abundance of CCR2, 4CL2, PAL3, and HCT26, and a lower abundance of CCoA-OMT (Additional file 7: Table S6). These results indicate that Cd exposure could trigger the lignification of CWs in peanut roots, and this was more pronounced in Silihong than in Fenghua 1. Increased lignin deposition induced by toxic metals also occurs in numerous plant species [47, 49–52]. Therefore, we can speculate that the higher CW lignification capacity of Silihong might be, at least partially, responsible for its higher Cd accumulation in CWs.

## Conclusions

**Fig. 6:**
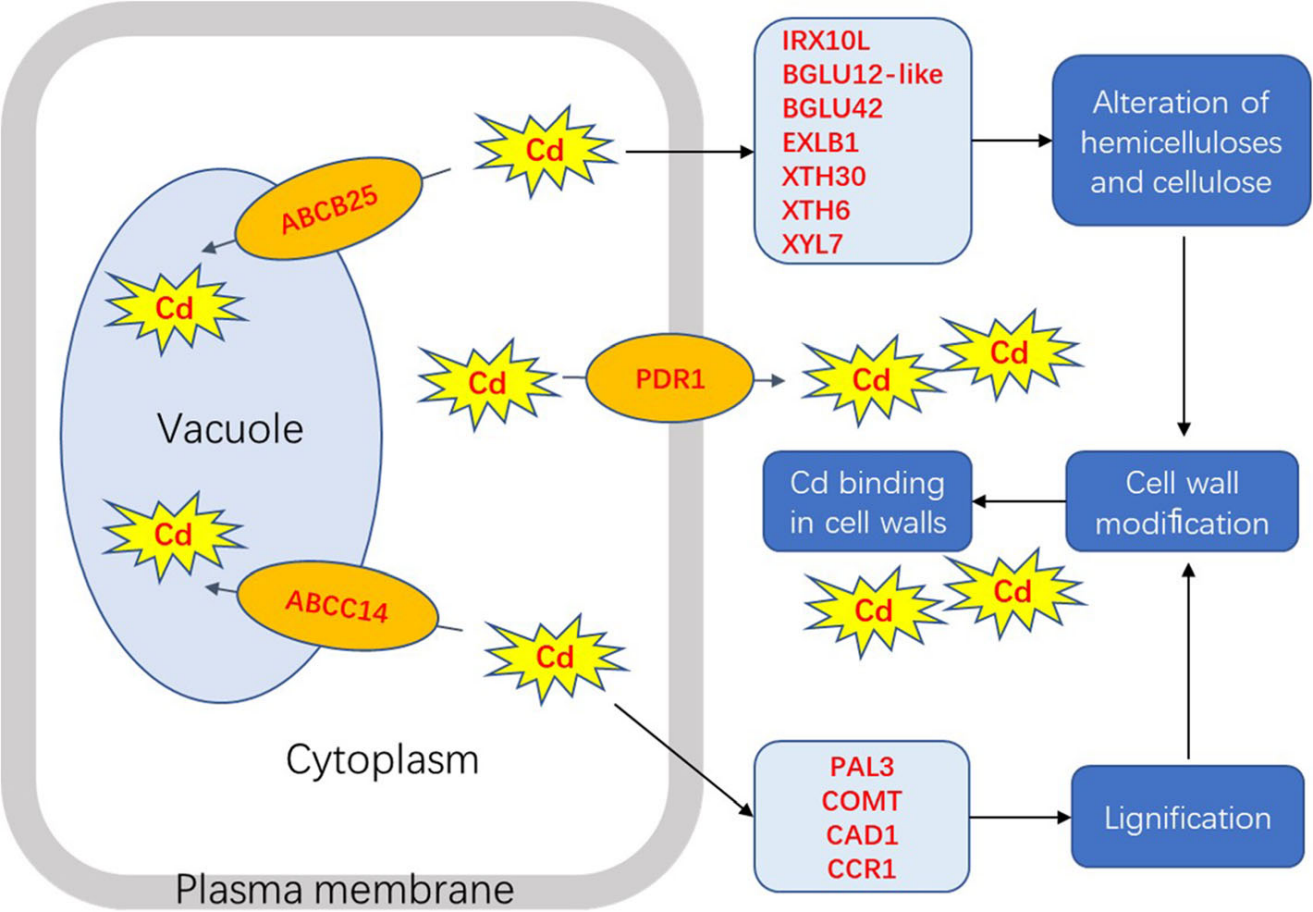
The putative model of cadmium detoxification in Silihong compared with Fenghua 1. DEPs highlighted with red colour indicate that they were up-regulated by Cd and showed significantly higher abundance in Silihong compared with Fenghua 1

This is a first systematic report of roots proteome changes between two peanut cultivars in response to Cd exposure. Totally, 375, 1762, 1276 and 771 DEPs were identified during F_Cd_/F_CK_, S_Cd_/S_CK_, F_CK_/S_CK_ and F_Cd_/S_Cd_ comparison, respectively. The two cultivars differed in the molecular mechanisms in response to Cd exposure. Silihong is more sensitive to Cd exposure than Fenghua 1 in terms of root proteomics. The up-regulation of ABCB25, ABCC14, ABCC2, PDR1 and V-ATPases by Cd exposure in Silihong might enhance vacuolar sequestration of Cd and its efflux from symplast to apoplast. The higher Cd accumulation in the root CWs in Silihong might be resulted from its higher capability of CW modification, in which many proteins such as IRX10L, BGLU12-like, BGLU42, EXLB1, XTH30, XTH6, XYL7, PAL3, COMT, CAD1, and CCR1 were involved. Thus, vacuolar sequestration and efflux of Cd as well as its adsorption in CW might be the principal mechanism of cadmium detoxification in Silihong (Fig. 6). We also confirmed that the higher abundance of ACA8 and ZIP1 in the roots of Silihong contributes to its higher capacity of root-to-shoot Cd translocation. These findings could provide novel insight for further understanding the molecular regulatory network of Cd accumulation in peanuts.

## Methods

### Plant materials and growth conditions

Based on our previous study [5], two peanut cultivars differing in seed Cd accumulation, Fenghua 1 (F, low Cd cultivar) and Silihong (S, high Cd cultivar) were used for this study. Seeds were commercially obtained from the Shandong Institute of Peanuts, Qingdao, China. After sterilized with 1% sodium hypochlorite for 10 min, seeds were sown in well-washed sand for germination. After emergence, seedlings with uniform size were transplanted into hydroponic pots (six plants per pot) containing 3.5 L of Hoagland nutrient solution (pH 5.8) [7]: 5 mM Ca (NO_3_)_2_, 5 mM KNO_3_, 1 mM KH_2_PO_4_, 1 mM MgSO_4_, 50 mM H_3_BO_3_, 4.5 mM MnCl_2_, 3.8 mM ZnSO_4_, 0.3 mM CuSO_4_, 0.1 mM (NH_4_)_6_Mo_7_O_24_ and 50 mM FeEDTA. For determination of Cd in plant tissues, seven-day-old seedlings were treated with 0, 0.2, 2 or 20 μM CdCl_2_ for 1 week. For other analyses, seven-day-old seedlings were treated with 0 (CK) and 2 μM CdCl_2_ (Cd) for 1 week. The experiment was arranged using a completely randomized design with three or four replications for each measurement. Plants were grown in a growth chamber as conditions previously described by Lu et al. [7].

### Root CW fractionation and measurement

Root samples (four replicates for each treatment) were collected from 0 (CK) and 2 μM Cd (Cd) for each cultivar. Root samples (0.5 g) were ground into powder with a mortar and pestle in liquid nitrogen and then homogenized with 7 mL 75% ethanol for 20 min in an ice-cold water bath. The extracted solutions were centrifuged with 1000×g at 4 °C for 10 min and the supernatant was removed. The precipitates were extracted twice with 7 mL of acetone, methanol:chloroform (1,1), and methanol, respectively, for 20 min each. The final crude CWs were freeze dried and weighed.

Fractionation of CW components were performed according to Zhong and Lauchli [53] and Wang et al. [25] with minor modifications. Pectin was extracted twice with 4 mL 0.5% ammonium oxalate buffer (including 1% KHB_4_) in a hot water bath (90–100 °C) for 10 min. The cooled solutions were centrifuged at 17,000×g for 10 min and the supernatants containing pectin were pooled together for each sample. The pellets were washed twice with ddH_2_O and then, they were further extracted twice with 4 mL 4% KOH (including 1% KHB_4_) at room temperature for 12 h. After centrifugation with 17,000×g for 10 min, the supernatant containing HC1 component was collected. Following the same method, HC2 was extracted twice with 4 mL 24% KOH (including 1% KHB_4_). Alkaline-insoluble cellulose was dissolved in 72% sulfuric acid at 25 °C for 1 h and then diluted 30-fold. The pH of the HC1 and HC2 supernatant was adjusted with acetic acid to 6.8–7.2 so that all the supernatants had the same volume.

The content of total sugar and uronic acid was determined for each fraction as the methods described by Wang et al. [25].

### Cd determination

After 7 days of Cd treatment, plant roots were immersed in 20 mM Na_2_-EDTA for 15 min, and then they were rinsed with deionized water three times. Seedlings for each treatments (three replicates) were dissected into roots and shoots. Thereafter, roots and shoots were dried to constant weight at 65 °C. The dried samples were weighed and digested in mixed acid [HNO_3_ + HClO_4_ (3:1, *v*/v)]. For CW Cd determination, the crude CWs were prepared for each treatment (four replicates) as the method mentioned above [53]. The dried CWs were digested with mixed acid [HNO_3_ + HClO_4_ (3:1, v/v)] after weighing. Cd concentrations were determined by atomic absorption spectrometry (WFX-110, Beijing Rayleigh Analytical Instrument Company, China) as previously described [8]. Translocation factors of Cd from the roots to shoots were calculated as the ratio of Cd concentrations in shoots to roots.

### Protein extraction and quantification

Root samples for iTRAQ-Seq (two biological replicates) were collected separately from plants treated with 0 (control) and 2 μM Cd (Cd) for each cultivar. A biological replicate contains a pool of six different plants. Approximately 1 g of each sample was grounded into powder in liquid nitrogen with 10% PVPP. The powder was transferred into Lysis buffer (8 M Urea and 40 mM Tris-HCl containing 1 mM PMSF, 2 mM EDTA and 10 mM DTT, pH 8.5), and sonicated on ice for 5 min. After centrifugation with 25,000 g at 4 °C for 20 min, the supernatant was treated to block the cysteine with 55 mM IAM in a dark room for 45 min. Then the supernatant was treated by adding 5 × volume of 10%TCA/acetone with 10 mM DTT to precipitate proteins at − 20 °C for 2 h. After centrifugation with 25,000 g at 4 °C for 20 min, the supernatant was discarded and the protein pellet was dried in the air for 5 min. Protein pellets were then dissolved in 200 μl 0.5 M TEAB, followed by centrifugation at 25,000 g for 20 min. The protein was then quantified using the Bradford method with bovine serum albumin (BSA) as the standard.

### Protein digestion, iTRAQ labeling and SCX fractionation

A total of 100 μg proteins for each sample were digested with trypsin gold (Promega, Madison, WI, USA) at 40:1 mass ratio at 37 °C for 12 h. After digestion, peptides were desalted using Strata X C18 column (Phenomenex) and vacuum-dried following the manufacturer’s protocol. The iTRAQ labeling of peptide was processed using iTRAQ reagent 8-plex kit following the manufacturer’s instructions. The eight samples including S_Cd__1, S_Cd__2, S_CK__1, S_CK__2, F_Cd__1, F_Cd__2, F_CK__1 and F_CK__2 were labeled with tags 113 and 114, 119, 121, 117, 118, 115 and 116, respectively, and incubated at room temperature for 2 h. For peptide fractionation, the strong cationic exchange (SCX) chromatography was carried out with a LC-20AB HPLC Pump system (Shimadzu, Kyoto, Japan) with an Ultremex SCX column (4.6 × 250 mm) as previously described [54].

### LC-ESI-MS/MS analysis

Each fraction was resuspended in buffer A (2% acetonitrile, 0.1% formic acid) and centrifuged at 20,000 rpm for 10 min. 10 μL supernatant was loaded on a 2 cm C18 trap column (200 μm inner diameter) and eluted onto a resolving 10 cm analytical C18 column (75 μm inner diameter) using a LC-20 AD nanoHPLC system (Shimadzu, Kyoto, Japan). The samples were loaded at 15 μL min^− 1^ for 4 min. A linear gradient from 2 to 35% solvent B (98% acetonitrile, 0.1% formic acid) was run over 44 min at 400 nL/min, and then followed by 2 min linear gradient to 80%, and maintained at 80% solvent B for 4 min, and finally returned to 2% in 1 min. Data acquisition was performed with a Triple TOF 5600 System (AB SCIEX, Concord, ON) fitted with a Nanospray III source (AB SCIEX, Concord, ON) and a pulled quartz tip as the emitter (New Objectives, Woburn, MA).

### Database search and protein quantification

The Proteome Discoverer software (Thermo Scientific) was used to convert the raw MS/MS data into MGF format files, which were searched against peanut genome database (ftp://ftp.ncbi.nlm.nih.gov/genomes/Arachis_duranensis/) using Mascot version 2.3.02 (Matrix Science, London, UK). At least one unique peptide was necessary for identification of a protein. The labeled peptides by isobaric tags for quantification analysis were performed using IQuant software as previously described [55]. To assess the confidence of the peptides, peptide-spectrum matches (PSMs) were pre-filtered at a PSM-level false discovery rate (FDR) of 1%. Thereafter, based on the “simple principle” (parsimony principle), the identified peptide sequences were assembled into a set of confident proteins. To control the rate of false-positive results at the protein level, a protein FDR at 1%, which was based on the selected protein FDR strategy, was estimated after protein inference (protein-level FDR ≤ 0.01). Quantitative protein ratios were weighted and normalized by the median ratio in Mascot. A protein with Bonferroni-corrected *P*-value < 0.05 and fold changes > 1.2 or < 0.833 was considered as being significant differentially expressed in the pairwise comparison.

Gene Ontology (GO) (http://www.geneontology.org) functional annotation of identified proteins were searched against the non-redundant protein database using Blast2GO program (https://www.blast2go.com/). Kyoto Encyclopedia of Genes and Genomes (KEGG) (http://www.genome.jp/kegg/pathway.html) database was adopted to categorize these identified protein species using Blastx/Blastp 2.2.24 software. Then, GO and KEGG pathway enrichment analysis of the DEPs were implemented with a *P*-value < 0.05.

### RT-qPCR analysis

Total RNA was extracted from three biological replicates of root samples of both peanut cultivars exposed to 0 and 2 μM CdCl_2_ treatments, respectively. Then the first strand cDNA was synthesized by Prime Script® RT reagent Kit (Takara, Dalian, China). The specific primers for RT-qPCR analysis were designed using Beacon Designer 7.0 software (Premier Biosoft International, USA), and *Actin* gene was set as the internal standard (Additional file 8: Table S7). RT-qPCR was conducted on an ABI7300 system (Applied Biosystems, Foster City, CA, USA) using SYBR® Green Master ROX (Takara) in a 20 μl reaction according to the method described by Yu et al. [56]. All reactions were performed in three technical replicates. Relative expression level was processed with the 2^-ΔΔ*C*T^ method.

## Additional files

Additional file 1: **Figure S1.** The repeatability analysis of data obtained from iTRAQ based on CV (coefficient of variation) analysis. (DOCX 644 kb)

Additional file 2: **Table S1.** List of Cd-induced differentially expressed proteins in roots of two peanut cultivars. (XLSX 3414 kb)

Additional file 3: **Table S2.** Significantly enriched GO terms for the DEPs. With *P*-value < 0.05 as cutoff. (XLSX 46 kb)

Additional file 4: **Table S3.** Most enriched GO terms for the DEPs. With *P*-value < 0.01 as cutoff. (XLSX 22 kb)

Additional file 5: **Table S4.** Differentially expressed proteins related to ion transporters. (XLSX 27 kb)

Additional file 6: **Table S5.** Differentially expressed proteins involved in cell wall metabolism in two peanut cultivars. (XLSX 21 kb)

Additional file 7: **Table S6.** Differentially expressed proteins involved in lignin biosynthesis. (XLSX 16 kb)

Additional file 8: **Table S7.** The primers used in RT-qPCR analysis. (XLSX 11 kb)

## Abbreviations

4CL: 4-coumarate-CoA ligase
ABC: ATP-binding cassette transporter protein
CAD: Cinnamyl alcohol dehydrogenase
CCoA-OMT: Caffeoyl-CoA O-methyltransferase
CCR: Cinnamoyl-CoA reductase
Cd: Cadmium
DEP: Differentially expressed protein
GO: Gene ontology
KEGG: Kyoto encyclopedia of genes and genomes
MTP: Metal tolerance protein
Nramp: Natural resistance-associated macrophage protein
OPT: Oligopeptide transporter
PDR: Pleiotropic drug resistance-type ABC transporter protein
PE: Pectinesterase
RT-qPCR: Reverse-transcription quantitative PCR
ZIP: Zinc-regulated transporter/Iron-regulated transporter-like Protein
