# Supplementary material for: Comparative proteomics analysis of peanut roots reveals differential mechanisms of cadmium detoxification and translocation between two cultivars differing in cadmium accumulation
Source: BMC Plant Biol. 2019 Apr 11;19:137. doi: 10.1186/s12870-019-1739-5 (PMC6458636; doi:10.1186/s12870-019-1739-5)
Supplement: Supplementary file 1 — Figure S1. The repeatability analysis of data obtained from iTRAQ based on CV (coefficient of variation) analysis. (DOCX 644 kb) [file 12870_2019_1739_MOESM1_ESM.docx]

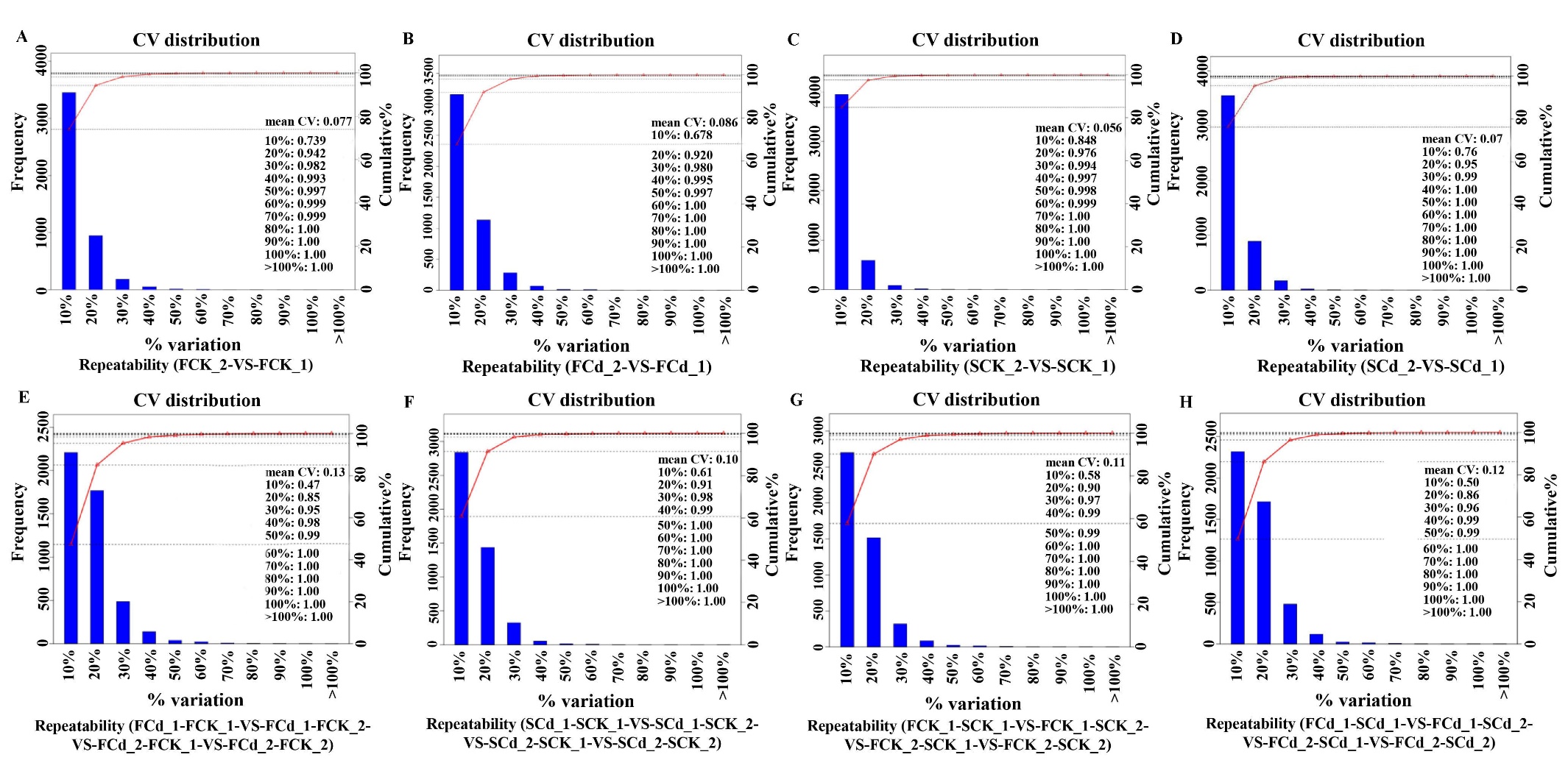


**Figure S1** The repeatability analysis of data obtained from iTRAQ based on CV (coefficient of variation) analysis. (A-D) indicated the repeatability analysis of two biological replicates, while (E-H) indicated the repeatability in different comparison groups. X-axis represented different variation levels, the left Y-axis represented the number of quantitative proteins at different variation level, and the right Y-axis represented the percentage that protein at a certain angle comprise quantified protein amount. CV is defined as the ratio of the standard deviation (SD) to the mean. The lower the CV, the better the reproducibility.
